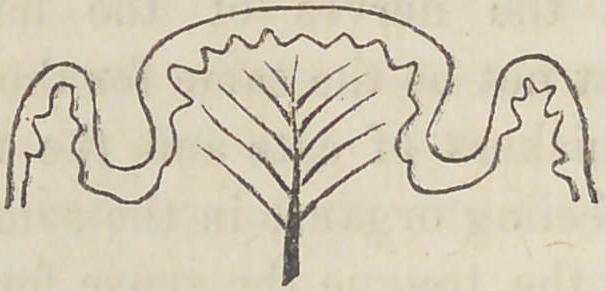# Do Gum Plates Injure Our Taste?

**Published:** 1867-06

**Authors:** A. Seiffert


THE
DENTAL REGISTER.

VOL. XXI.] JUNE, 1867. [NO. 6.

### Original Communications.

### DO GUMPLATES INJURE OUR TASTE?

FROM THE "ZAHNARZT," BY A. SEIFFERT.

IT is a common and not yet established complaint of those individuals, who use artificial teeth on suction plates, that their taste is more or less injured. If we would prove to them, with the whole physiological literature, that the *tongue* is the only seat for the taste, and if we would prove these different experiments, they would simply reply to us, what we so often hear from intelligent ladies: "*But sir, since I use the artificial set I have no, or very little taste for my aliments; and in order to bring them eatable to the table, I must test them without the artificial teeth.*"

The savant professors do not cook aliments on their writing desks or section tables, and all their arguments are idle against a single fact. An esteemed friend wrote once to me, I shall bring a solution of quinine to the patient's tongue, without letting the tongue touch the palate, and he would experience no taste at all till he closes the mouth, or till he brings his tongue in contact with the quinine. To him I say: "Bring our so generally used composition of morphine, arsenic and creosote *carefully*, without great pressure, on the tongue, and you will feel a weak chemical action, which is realized by the sensory reflection of the ramus lingualis trigemini, but you will feel nothing of a taste, till the tongue is pressed against the palate." The chemical action is felt on the *mucous membrane of the lip*, as a burning sensation *at least*, with the same vigor, without the interposition of any gustatory nerve.

If we reduce now the taste, to that which signifies the basis of each sense, viz.: the *feeling*, then we must confess that an organ, whose nerves lie so close together, as is the case with the tongue, can give us better proofs than an organ whose *nerve territory* is more extended. But the *feeling* is produced by a very simple apparatus, which in the organ for taste is only a more complicated repetition. Let us investigate now this question more particularly.

We find in the TASTE CORPUSCLES an elevation of the skin, which includes a cellular organ, containing a neutral fluid, composed of oxygen and hydrogen. In the middle of the organ is that *end of the nerve*, which rises from the respective sensible nerve, and which ramifies with single points, whose end ramifications are not more visible with the microscope. From the inside of the included cellular organ are hanging down fine *lappets* corresponding with the points; but they do *not touch* the end points of the sensitive nerves. The slightest pressure on the outside skin brings the lappets in contact with the cellular organ, and so causes the sense of taste, since under such circumstances a *circulation of an electric fluid* is created, which is the *taste feeling*. We are now arrived at the simple phenomenon of electro-magnetism.

We have once already remarked, that in the nerve-tubes even of the ax-cylinders, the triangular form of them contains that vis vitalis, after which we have been hunting so long, and which exists as the gas of *nitrogen*. After closer investigation, we have found that there are also *round* ax-cylinders. We must, after our physical and chemical observations conclude, that these *round* ax-cylinders contain principally *carbogen*, which is to be found in the above named *lappets* as the end-points of the *sympathetic nerve.* If by pressure both come in contact, then the conduit is established and indeed a *double* one. Whilst the *direct currents to the brain*, through the telegraph wire of the *sensitive nerve* is produced by our will, an other *indirect current* (earthen current) takes place by the sympathicus. The investigations of *Schiff* and *Brown-Sequard* have stated, that the GRAY SUBSTANCE CONDUCTS THE IMPRESSIONS OF THE SENSE, WITHOUT BEING ITSELF SENSITIVE. *Schiff* calls therefore the gray substance the *æstheosodic substance*, and he says, that the common sensations are conducted through that gray substance, but that the feeling of touch is carried to the brain in the *white* substance. But not the gray *substance alone*, the fibres of the white substance, which penetrate them are also æstheosodic (*i.e.* conducting the feeling, without being self-feeling). But we know even those fibres as leaders of the so-called reflex-feelings. By further exploration, why causes act on one nerve differently to what they act on another, we find (after Setschenow) the remarkable circumstance, that *chemical* irritations depress, but that *tactile* irritations improve the faculty of reaction of the brain. This must be produced by special actions to the one, and to the other side. We know also, that *that acids produce a contraction, alkalies an expansion.* If we imagine that by contraction of the taste corpuscles containing fluids, produced by a quick imbibition of an outside applied *acid* substance, the same result is gained as if by a mechanical pressure, with the *only* difference that the CONTRACTED LAPPETS are compelled to touch the *sensible nerve-fibres*; and that again externally applied *alkalies* produce an expansion, a stretching out of the SENSIBLE FIBRES, which again create a *feeling*, then we have already *three modifications* of the same feeling, which we are able to discern as feeling of pressure, and feeling of sour and alkalic re-agents. And there is yet a fourth and fifth impression of feeling, namely, of *warm* and *cold.* This we can easily imagine, is set in motion differently from the above described both kinds, since here none of the two nerve-ends are affected, but a common expansion, and a common contraction is only produced by the influence of the agents.

We have followed so far the straight line, but now let us pass a side-walk, which notwithstanding will lead us to the main street again.

It is a fixed state, that the *normal leading* of the impressions are performed by the longitudinal fibres of the *hint cerebral trunks* (crura cerebri), to-wit.: the trunks from the *same side*; and only when the one side is paralyzed the leading is transported to the *other side.* It is a further fact, that when the half of the medulla is separated diametrically, the skin of the same side below the separated part becomes hyperæsthesied, whilst on the other side a depressed feeling, an *anæsthesia* is caused. From that we conclude that a *provisoric action of other nerve fibres* takes place instead of those that came out of action, and that further a *modified action* arises as soon as the balance of the two halves in one or the other way is injured.

The above bespoken kind of *taste organs* (corpuscles), we find on the tongue as FUNGIFORME PAPILLAS, and when we look closer we find that the points and the sides of the tongue are mostly occupied by them. But here dominates the *nervus lingualis trigemini,* and therefore we are already able to affirm that this nerve represents the *sensitive* nerve of the tongue, which informs our soul of the form, magnitude, gravity, hardness or softness of a substance, and which gives notice of temperature, of sour and alkalic properties, a sharp and scratching taste.

We will notice here a remarkable property of the sensitive nerve. When we put our tongue in warm water of 41-42 R° or in ice, then we lose the property of taste, or this is spoiled for some moments. The importance of this interposed remark is easily to be understood, when we remember the anæsthesia produced by cold; and therefore the possibility of painless Dental operations is only then of a result, when the refrigerating process penetrates.

Instead of pursuing the taken course, and describing the second kind of skin-nerves, and then comparing the adequate nerves of the tongue with them, we will take the adverse course, and will bring now the second form of papillar nerve-end of the tongue before our knowledge, and then only the equal form of the skin-nerves.

Whilst the first kind rises up in form of *papillæ*, the other kind elevates FILIFORM, but the arrangement of the parts is notwithstanding the same. The filiform kind have also the lappets, in whose centre the *ramifications* of the sensible *fili* are visible with their simple ending points; and we could pass this form if it would not be necessary to know their function. The whole form of them let us suppose, corresponds with the nerves of the hair-follicles of the skin. Should that not be the same for the tongue? Certainly! for, as the whiskers of cats and the tentacles of many insects are their feeling organs, in the same manner accommodate the fili of the tongue the sense for the taste through their sensibility, which no doubt must come out *more subtile*, as in the form of papillæ. But they have another purpose. We know by experience, that terrible impressions cause *hair erecting* upon us, and that an excited action of the hair-follicles, which produces the so-called goose-skin, is not only to be felt, but also to be seen. This demonstrates to us, that those filiform elevations are in connection with the *motoric nerve of the tongue, i. e.* with the nervus sublingualis.

We have now gone so long around the pap, that it is not hot any more, but easily to be understood; and we turn now to those nerves, to which we attribute the veritable sense of taste, to the nervi *glosso-pharyngei.*

Strange, why the *glosso-pharyngeus* (tongue-throat), why not simply nervus pharyngeus (tongue-nerve)? Do you not know, that it ramifies not only in the hind part of the dorsal tongue, (who is said to possess the finest taste), but also in the soft palatine? This must have its peculiar meaning. And in fact as many authors, that have laid the sense of taste in the tongue, as many have placed it in the palatine, viz.: Biffi, Budge, Klaatsch, Kornfeld, Morganti, I. Muller, Schirmer, Stanius, Stich, Valentin, Veriere, and to make the dozen full, the Author.

We are compelled to take this nerve also like the other two in closer consideration, and especially the peculiar organs in which one part of its fibres determinate—I mean the *papillæ vallatæ.*

The *papillæ vallatæ* are a composition of the above named two forms, but so that the *fungiform papillæ* appear to be larger, and surrounded by a circular wall of *filiform papillæ.* A section of such a papillæ would represent as follows:

The wall contains ending fibres of the sublingualis like the filiform papillæ. It incloses a fortification, a reservoir, and with the end-fibres of the motoric sublingualis it is able to change the reservoir into a circular canal, in which the fluid is inclosed. By doing this through pressure comes forth not only an electric current in the ramifications of the glosso-pharyngeus, but also a real battery through the association of the wall-edges, of which the result is *the feeling of taste.*

Therefore we find, that the taste-feeling represents a combination of the irritative and sensible system, whose action is only increased by the peculiar form of the organs, and that also there, where similar nerves *are standing near together,* similar results are gained, and therefore every part of the tongue, and every part of the *soft and hard palate* must support more or less the taste-feeling.

This can be proved by the following experiment: If you moisten the upper and under lip with saliva, and if you touch one or the other lip with a strong tasting substance, but carefully avoiding the touching of the tongue, then you will feel the oftener you open and close the lips (that is the more the substances are diffused over the nerves) a dull taste of the substance; therefore the lips are also organs for taste.

We draw, in conclusion, your attention to a positive law, in the doctrine of electricity, namely: THE INTENSITY OF FEELING GROWS WITH THE SUM OF THE IN THE SAME TIME EXCITED NERVE-FIBRES, AS THE SINGULAR IMPRESSIONS FOR CONCEPTION APPEAR, SO TO SAY, TO SUM UP. This alone would be enough to shake the opinion, that *artificial plates do not injure the taste*, if not a second law would spring up, which is much stronger yet for injury of the taste.

On the side of the tongue, we have a quantity of galvanic apparatus, which collectively are, what we will call taste; and we have on both sides of the palatine, a quantity of weaker apparatus with similar structure and action, by which the taste is accommodated; but the partial proof of those organs shows us the feeling of taste, but not the taste itself. This is then felt, when the bolus, mixed with the mucus of the sour mucilaginous glandula, and the alkalie pituitary gland, comes in contact with many conductors of the tongue and the palate; fluids are conductors of themselves. When these conducting faculties are interrupted by electro-positive materies, viz.: caoutchouc, the natural taste MUST be spoiled.

We have already stated that a *modified action* takes place, as soon as the equilibrium of the taste-producing nerves is spoiled. *That part of taste*, which the tongue-nerves themselves lose through their weak conducting power, as the palate is partially covered by the caoutchouc, can not immediately *be entirely* regulated, time only brings in *some degree* compensation, but never as it was before. Do THEREFORE GUM-PLATES INJURE THE TASTE?

## Figures and Tables

**Figure f1:**
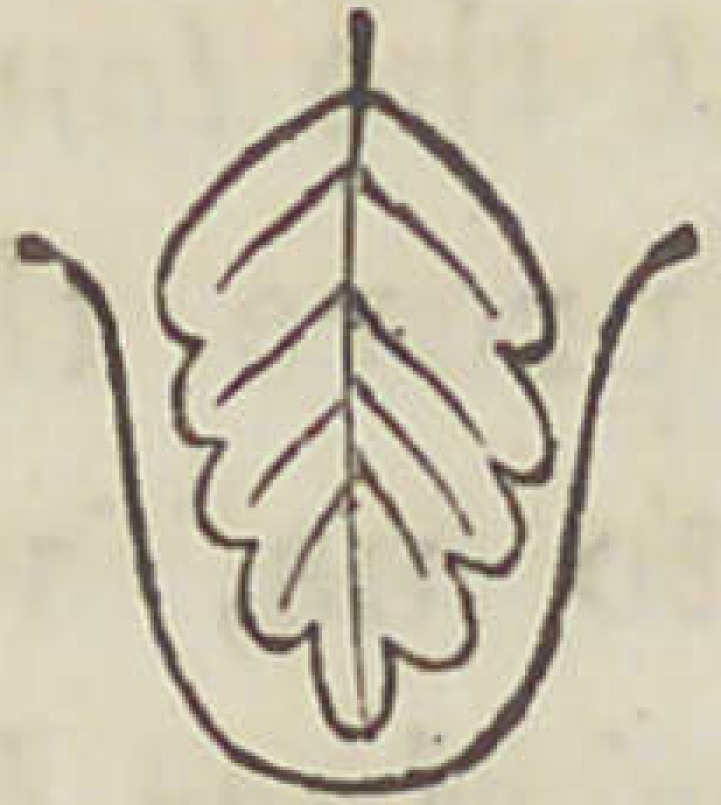


**Figure f2:**